# Factors Associated With Avoiding Health Care Among Community-Dwelling Medicare Beneficiaries With Type 2 Diabetes

**DOI:** 10.5888/pcd17.200148

**Published:** 2020-10-15

**Authors:** Boon Peng Ng, Jacqueline B. LaManna, Samuel D. Towne, Brian C. Peach, Qing He, Chanhyun Park

**Affiliations:** 1 College of Nursing, University of Central Florida; 2 Disability, Aging, and Technology Cluster, University of Central Florida; 3 Department of Health Management & Informatics, University of Central Florida; 4 Department of Environmental and Occupational Health, School of Public Health, Texas A&M University; 5 Southwest Rural Health Research Center, Texas A&M University; 6 Center for Population Health and Aging, Texas A&M University; 7 Department of Statistics and Data Science, University of Central Florida; 8 Department of Pharmacy and Health Systems Sciences, School of Pharmacy, Northeastern University

## Abstract

**Introduction:**

Health care avoidance by Medicare beneficiaries with chronic conditions such as type 2 diabetes can result in adverse health and economic outcomes. The objective of this study was to describe factors associated with choices to avoid health care among Medicare beneficiaries with type 2 diabetes.

**Methods:**

We used a survey-weighted logistic model and the nationally representative 2016 Medicare Current Beneficiary Survey to analyze data on 1,782 Medicare beneficiaries aged ≥65 with type 2 diabetes, to examine associations between Medicare beneficiaries’ decisions to avoid health care and multiple factors (eg, dissatisfaction with information given by providers, health problems that should have been discussed with providers but were not, worry about health more than other people their age).

**Results:**

Of our study sample, 26.1% reported they avoid health care. Five factors were associated with avoiding health care: delaying care (vs not) because of costs (adjusted odds ratio [aOR] = 2.06; *P* = .005); having health problems that should have been discussed with providers but were not (vs having discussions) (aOR = 1.50; *P* = .04); worrying (vs not) about health more than other people their age (aOR = 2.13; *P* < .001); self-reporting “other” minority race (vs non-Hispanic White) (aOR = 2.01; *P* = .006); and education levels. Participants with less than a high school diploma (aOR = 1.95; *P* = .001) and participants with a high school diploma only (aOR = 1.49; *P* = .049) were more likely than participants with an education beyond high school to report avoiding health care.

**Conclusion:**

Approximately 1 in 4 Medicare beneficiaries with type 2 diabetes avoid health care. We found inequities in care-seeking behavior by race/ethnicity and education level. Health care perceptions and lack of appropriate discussion of health care concerns with health care providers are also associated with this behavior. Clinical interventions (eg, improved patient–provider communication) and educational outreach are needed to decrease the numbers of Medicare beneficiaries who avoid health care.

**Summary**

**What is already known on this topic?**

Factors associated with choosing to avoid health care have been investigated in the adult population; however, little is known about factors associated with this behavior among older adults with diabetes.

**What is added by this report?**

More than 25% of Medicare beneficiaries with type 2 diabetes reported avoiding health care. For this population, after we accounted for covariates, factors such as education, race/ethnicity, costs, health care perceptions, and patient–provider communication were associated with avoiding health care.

**What are the implications for public health practice?**

Screening for avoiding health care, better patient–provider communication, and educational outreach efforts are needed to encourage older adults with diabetes to seek health care.

## Introduction

Older adults living with type 2 diabetes are faced with a condition that is complex to manage, necessitating frequent interactions with health care professionals (1). Failure to seek recommended care (eg, checkups or screenings) can make these older adults particularly vulnerable to many diabetes-related complications. Although barriers to health care are well-described in the literature, additional study is needed to understand why people with chronic conditions choose to avoid health care, especially among Medicare beneficiaries who have type 2 diabetes (2–5).

Health care avoidance occurs when a person or population “distances itself from utilization of preventive health services, treatment seeking, and treatment adherence” (4). Health care avoidance is associated with an interplay of demographic, geographic, and psychologic factors, with the psychologic factors being most predictive of the decision to avoid necessary health care (2,3,5). Although cost of care is a factor in avoiding health care, individual factors, such as low health self-efficacy, poor previous experiences with medical providers, fear or dislike of medical procedures, discomfort with body examination, and fear of a serious diagnosis also contribute to these decisions (2,3,5,6).

A national survey conducted in 2018 found that approximately 40% of Americans skip recommended tests or treatments, and approximately 44% fail to seek care when sick or injured because of costs (6). Of those who delayed or skipped care, approximately 86% had insurance coverage (6). These findings have implications for people with chronic illnesses such as diabetes, which affects approximately 25% (approximately 10 million) of people aged 65 or older in the United States (7). Delayed treatments or skipped medications among people with diabetes can lead to increased risk and severity of complications (eg, amputation, kidney failure, blindness, cardiovascular disease), which may result in greater use of the emergency department and longer inpatient stays. According to the Centers for Medicare & Medicaid Services, Medicare spent an estimated $42 billion on diabetes care in 2016 (8), and approximately 40% of older adults with diabetes had 3 or more comorbidities (1).

Type 2 diabetes accounts for 90% to 95% of all diabetes cases (7). Type 2 diabetes often can be delayed or even prevented through maintenance of a healthy lifestyle and participation in evidence-based lifestyle modification programs (9). Diabetes-related complications may be reduced through patient-centered disease management programs (10). Understanding factors associated with people choosing to avoid health care is important to develop targeted prevention strategies. In the absence of such knowledge, clinicians may not effectively engage with this at-risk population, who can benefit from preventive, treatment, and management programs. Engagement in these programs can prevent burdensome and costly diabetes-related complications. The objective of our study was to describe factors associated with decisions to avoid health care among Medicare beneficiaries aged 65 or older with type 2 diabetes.

## Methods

We analyzed data from the 2016 Medicare Current Beneficiary Survey Public Use File from the Centers for Medicare & Medicaid Services, which is a continuous and multipurpose survey of a representative, national sample of Medicare beneficiaries (11). The data set includes data only on community-dwelling beneficiaries, excluding data on beneficiaries living in long-term care facilities (11). It includes information on Medicare enrollment, sociodemographic characteristics, health conditions, and health care access. The data set is a shorter version of the original Medicare Current Beneficiary Survey; it includes fewer variables and categorizes some variables more broadly (11). We accessed and analyzed the data set in 2019.

Our study population consisted of 1,782 Medicare beneficiaries aged 65 or older with type 2 diabetes. We identified the presence of type 2 diabetes by examining data from 2 items in the 2016 Medicare Current Beneficiary Survey Public Use File (Appendix): 1) “Has a doctor ever told you that you had any type of diabetes, including sugar diabetes, high blood sugar, borderline diabetes, prediabetes, or pregnancy-related diabetes/borderline diabetes or prediabetes?” and 2) “Please tell which type of diabetes the doctor said you have.”

### Measures

For the outcome variable, we identified Medicare beneficiaries with type 2 diabetes whose response to the following question indicated if they would avoid health care: “You will do just about anything to avoid going to the doctor. True or false?”

For key factors of interest, we included the following variables: delayed seeking care because of costs (yes/no), dissatisfaction with ease of getting to health care providers (a 5-point Likert scale), dissatisfaction with information given by health care providers (a 5-point Likert scale), health problems that should have been discussed with health care providers but were not (a 5-point Likert scale), and worried about health more than other people their age (true/false) (Appendix).

We recoded Likert-scaled variables into broader categories to ensure adequate sample sizes for reliable estimates. For example, participants were asked to rate on a 5-point Likert scale their agreement with the statement on health problems that should have been discussed with providers but were not: 1 = strongly agree, 2 = agree, 3 = neither agree nor disagree, 4 = disagree, and 5 = strongly disagree. We recoded the variable by combining response levels of 1 and 2 together (strongly agree and agree) and 4 and 5 together (disagree and strongly disagree), and we created a categorical variable with 3 categories: 1 = strongly agree/agree (yes), 2 = disagree/strongly disagree (no), and 3 = neither agree nor disagree. We created 2 dichotomous variables for dissatisfaction with ease in getting to health care providers and information given by health care providers: 1 = very dissatisfied/dissatisfied (yes); 0 = very satisfied/satisfied (no).

The use of covariates for the analysis was guided by previous research (2,3,5). Covariates were sex (male or female), age group (65–74 or ≥75), race/ethnicity (non-Hispanic White, non-Hispanic Black, Hispanic, or other), education level (<high school diploma, high school diploma only, or >high school diploma), annual household income (<$25,000 or ≥$25,000), marital status (married, widowed, divorced/separated, or never married), residing area (metropolitan or nonmetropolitan), living status (alone or not alone), body mass index (in kg/m^2^; underweight, <18.5, healthy weight, 18.5 to <25.0, or overweight/obese, ≥25.0), general health status (excellent/very good, good, or fair/poor), and 7 comorbidities (hypertension/high blood pressure, myocardial infarction/heart attack, stroke/brain hemorrhage, emphysema/asthma/chronic obstructive pulmonary disease, rheumatoid arthritis, depression, and urinary incontinence). Additionally, we included covariates on functional status, both activities of daily living (ADLs) and instrumental activities of daily living (IADLs). ADLs are skills required for such everyday activities as bathing, dressing, toileting, transferring to chairs, walking, and eating, whereas IADLs are skills that require more complex planning and thinking, such as managing money, shopping, using the telephone, housekeeping, and preparing meals. We recoded IADL/ADL limitations as no limitations, only IADL limitations, 1 or 2 ADL limitations, or 3 or more ADL limitations.

### Statistical analyses

We calculated proportions for each measure overall and then stratified data by whether health care was avoided. We compared differences in proportions of respondents avoiding health care and not avoiding health care by using Wald χ^2^ tests. We used a logistic model adjusted for sociodemographic characteristics and comorbidities to examine associations between these factors and the choice to avoid health care. Results were considered significant at *P* < .05.

All analyses applied survey weights to account for the complex survey design. We used a subgroup/domain analysis to ensure the accuracy of estimates. We performed all analyses by using SAS Enterprise Guide version 6.1 (SAS Institute Inc) and Stata/IC version 11.2 (StataCorp LLC).

## Results

Among 1,782 Medicare beneficiaries with type 2 diabetes in our study sample, 465 (26.1%) reported that they would avoid health care (Table 1). The proportion of respondents who reported avoiding health care was significantly greater than the proportion who reported not avoiding health care among the following groups: women (55.1% vs 46.7%), Hispanic respondents (15.6% vs 8.8%), respondents of “other” race/ethnicities (12.5% vs 6.8%), respondents with less than a high school diploma (29.2% vs 15.9%) or with a high school diploma only (37.1% vs 31.8%), and respondents with less than $25,000 in annual household income (42.5% vs 32.6%). Respondents who reported avoiding health care had lower levels of functional skills (eg, 1 or 2 ADL limitations, 26.3% vs 21.0%) and reported worse general health status than respondents without this care-seeking behavior (eg, fair/poor health, 34.2% vs 25.4%). Compared with respondents who did not avoid health care, respondents who avoided health care indicated higher levels of cost-based decision making (15.5% vs 7.0%), health problems that should have been discussed with providers but were not (14.2% vs 8.3), and greater worry about health than others their age (35.9% vs 17.8%). Although not significantly different according to Wald χ^2^ tests, 5.7% and 6.8% of beneficiaries choosing to avoid health care reported dissatisfaction with ease of getting to providers and dissatisfaction with information given by providers compared with 3.4% and 4.3% of respondents who did not avoid health care, respectively.

**Table 1:** Characteristics of Medicare Beneficiaries Aged ≥65 With Type 2 Diabetes, by Whether They Avoid Health Care, Medicare Current Beneficiary Survey Public Use File, 2016^a^

Variable	Total	Avoid Health Care^b^	Do Not Avoid Health Care^b^	*P* Value^c^
**N, sample size**	1,782	465	1,317	—
**Weighted estimated no. (%) of beneficiaries**	7.5 million (100)	1.9 million (26.1)	5.5 million (73.9)	—
**Sociodemographic Characteristics^d^**				
**Age group, y**				
65–74	62.1	63.1	61.8	.64
≥75	37.9	36.9	38.2	
**Sex**				
Female	48.9	55.1	46.7	.008
Male	51.1	44.9	53.3	
**Race/ethnicity**				
Non-Hispanic White	69.6	60.6	72.7	.002
Non-Hispanic Black	11.5	11.3	11.6	
Hispanic	10.6	15.6	8.8	
Other	8.3	12.5	6.8	
**Marital status**				
Married	58.3	58.1	58.3	.28
Widowed	22.1	24.4	21.3	
Divorced/separated	14.1	13.7	14.2	
Never married	5.6	3.9	6.2	
**Education**				
<High school diploma	19.4	29.2	15.9	<.001
High school diploma only	33.2	37.1	31.8	
>High school diploma	47.4	33.7	52.2	
**Annual household income, $**				
<25,000	35.2	42.5	32.6	.001
≥25,000	64.8	57.5	67.4	
**Residence**				
Nonmetropolitan area	20.6	24.9	19.0	.08
Metropolitan area	79.4	75.1	81.0	
**Living status**				
Not alone	73.7	74.4	73.5	.70
Alone	26.3	25.6	26.5	
**Comorbidities and Health Status^d^**				
**Hypertension/high blood pressure**				
No	17.4	14.5	18.5	.06
Yes	82.6	85.5	81.5	
**Myocardial infarction/heart attack**				
No	84.8	84.1	85.1	.68
Yes	15.2	15.9	14.9	
**Stroke/brain hemorrhage**				
No	88.4	88.5	88.4	.94
Yes	11.6	11.5	11.6	
**Emphysema/asthma/chronic obstructive pulmonary disease**				
No	79.3	77.8	79.9	.36
Yes	20.7	22.2	20.1	
**Rheumatoid arthritis**				
No	83.7	78.8	85.4	.005
Yes	16.3	21.2	14.6	
**Depression**				
No	74.2	73.7	74.3	.83
Yes	25.8	26.3	25.7	
**Urinary incontinence**				
No	57.0	53.8	58.1	.17
Yes	43.0	46.2	41.9	
**Body mass index, kg/m^2^**				
Underweight (<18.5)	0.3	0.6	0.3	.65
Healthy (18.5 to <25.0)	18.3	18.4	18.3	
Overweight/obese (≥25.0)	81.3	81.0	81.4	
**IADL/ADL limitations**				
No limitations	54.3	46.5	57.1	.006
Only IADL limitations	11.1	12.1	10.8	
1 or 2 ADL limitations	22.4	26.3	21.0	
≥3 ADL limitations	12.2	15.1	11.2	
**General health status**				
Excellent/very good	35.4	26.8	38.4	.001
Good	36.9	39.0	36.2	
Fair/poo	27.7	34.2	25.4	
**Key Factors of Interest^d^**				
**Delayed seeking care because of costs**				
No	90.8	84.5	93.0	<.001
Yes	9.2	15.5	7.0	
**Dissatisfaction with ease of getting to health care providers**				
No	96.0	94.3	96.6	.07
Yes	4.0	5.7	3.4	
**Dissatisfaction with information given by health care providers**				
No	95.0	93.2	95.7	.10
Yes	5.0	6.8	4.3	
**Health problems that should have been discussed with health care providers but were not**				
No	28.8	21.7	31.3	<.001
Neither yes nor no	61.4	64.2	60.4	
Yes	9.8	14.2	8.3	
**Worried about health more than others their age**				
False	77.5	64.1	82.2	<.001
True	22.5	35.9	17.8	

Abbreviations: —, not assessed; ADL, activity of daily living; IADL, instrumental activity of daily living.

a Categories determined by asking a single question: “You will do just about anything to avoid going to the doctor. True or false?”

b Percentages may not total 100% because of rounding.

c Wald χ^2^ tests used to compare characteristics of beneficiaries by whether they avoided health care.

d Data shown are percentages, unless otherwise noted.

Respondents who indicated “other” race/ethnicity were 2.01 (95% CI, 1.23–3.30; *P* = .006) times more likely than non-Hispanic White respondents to avoid health care (Table 2). Respondents with less than a high school diploma (adjusted odds ratio [aOR] = 1.95; 95% CI, 1.32–2.90; *P* = .001) and respondents with a high school diploma only (aOR = 1.49; 95% CI, 1.00–2.23; *P* = .049) were more likely than respondents with more than a high school diploma to avoid health care. Respondents who delayed seeking care because of cost were twice as likely to avoid health care (aOR = 2.06; 95% CI, 1.25–3.40; *P* = .005) as respondents who did not delay care because of cost. Respondents who had health problems that should have been discussed with providers but were not were also more likely to avoid health care compared with those able to discuss their health problems (aOR = 1.50; 95% CI, 1.02–2.21; *P* = .04). Respondents who worried about their health more than others their age were twice as likely to avoid health care (aOR = 2.13; 95% CI, 1.49–3.04; *P* < .001) as those who did not have such worries.

**Table 2:** Factors Associated With Avoiding Health Care Among Medicare Beneficiaries Aged ≥65 With Type 2 Diabetes, Medicare Current Beneficiary Survey Public Use File, 2016^a^

Factor	Adjusted Odd Ratio^a^ (95% CI)	*P* Value
**Sociodemographic Characteristics**		
**Age group, y**		
65–74	1 [Reference]	
≥75	0.92 (0.69–1.21)	.53
**Sex**		
Female	1 [Reference]	
Male	0.80 (0.58–1.11)	.18
**Race/ethnicity**		
Non-Hispanic White	1 [Reference]	
Non-Hispanic Black	0.87 (0.56–1.36)	.54
Hispanic	1.24 (0.74–2.06)	.41
Other	2.01 (1.23–3.30)	.006
**Marital status**		
Married	1 [Reference]	
Widowed	0.87 (0.58–1.31)	.51
Divorced/separated	0.77 (0.45–1.34)	.36
Never married	0.52 (0.18–1.46)	.21
**Education**		
>High school diploma	1 [Reference]	
High school diploma only	1.49 (1.00–2.23)	.049
<High school diploma	1.95 (1.32–2.90)	.001
**Annual household income, $**		
≥25,000	1 [Reference]	
<25,000	1.07 (0.75–1.54)	.69
**Residence**		
Metropolitan area	1 [Reference]	
Nonmetropolitan area	1.37 (0.90–2.09)	.14
**Living status**		
Not alone	1 [Reference]	
Alone	1.05 (0.75–1.48)	.77
**Comorbidities and Health Status**		
**Hypertension/high blood pressure**		
No	1 [Reference]	
Yes	1.23 (0.87–1.74)	.23
**Myocardial infarction/heart attack**		
No	1 [Reference]	
Yes	0.89 (0.60–1.33)	.57
**Stroke/brain hemorrhage**		
No	1 [Reference]	
Yes	0.82 (0.56–1.20)	.30
**Emphysema/asthma/chronic obstructive pulmonary disease**		
No	1 [Reference]	
Yes	1.04 (0.78–1.40)	.77
**Rheumatoid arthritis**		
No	1 [Reference]	
Yes	1.21 (0.85–1.72)	.28
**Depression**		
No	1 [Reference]	
Yes	0.75 (0.52–1.09)	.13
**Urinary incontinence**		
No	1 [Reference]	
Yes	1.00 (0.74–1.35)	.99
**Body mass index, kg/m^2^**		
Underweight (<18.5)	1 [Reference]	
Healthy (18.5 to <25.0)	0.40 (0.05–3.11)	.38
Overweight/obese (≥25.0)	0.41 (0.05–3.17)	.39
**IADL/ADL limitations**		
No limitations	1 [Reference]	
Only IADL limitations	1.09 (0.68–1.77)	.71
1 or 2 ADL limitations	1.24 (0.90–1.70)	.19
≥3 ADL limitations	1.09 (0.63–1.87)	.76
**General health status**		
Excellent/very good	1 [Reference]	
Good	1.28 (0.87–1.89)	.21
Fair/poor	1.03 (0.60–1.76)	.93
**Key Factors of Interest**		
**Delayed seeking care because of costs**		
No	1 [Reference]	
Yes	2.06 (1.25–3.40)	.005
**Dissatisfaction with ease of getting to providers**		
No	1 [Reference]	
Yes	1.21 (0.66–2.21)	.53
**Dissatisfaction with information given by providers**		
No	1 [Reference]	
Yes	1.14 (0.56–2.31)	.72
**Health problems that should have been discussed with providers but were not**		
No	1 [Reference]	
Neither yes nor no	1.27 (0.94–1.71)	.12
Yes	1.50 (1.02–2.21)	.04
**Worried about health more than others their age**		
False	1 [Reference]	
True	2.13 (1.49–3.04)	<.001

Abbreviations: ADL, activity of daily living; IADL, instrumental activity of daily living.

a Avoidance of health care was determined by asking a single question: “You will do just about anything to avoid going to the doctor. True or false?”

## Discussion

Little is known about the characteristics of Medicare beneficiaries with reported type 2 diabetes who choose to avoid health care, nor about underlying factors associated with choosing to do so. In our study, more than 25% of insured, community-dwelling Medicare beneficiaries aged 65 or older with type 2 diabetes reported they would avoid going to the doctor. Older adults with diabetes are at risk of potentially preventable acute and chronic diabetes-related complications that could result in unplanned hospitalizations, poor quality of life, and even death (12,13). Consistent with the Behavioral Model of Healthcare Service Use (14,15), we showed that choosing to avoid health care among Medicare beneficiaries with type 2 diabetes is multifactorial, and a result of individual, economic, health care provider, and system-level factors.

Our findings, in general, are consistent with the findings of studies that focused on factors associated with avoiding health care, although those studies differed from ours in populations and settings. Kannan and Veazie found that approximately 36% of US adults aged 18 or older avoided physician visits (2). Although this percentage is higher than our estimate (26% of Medicare beneficiaries with type 2 diabetes avoiding health care), the finding is not surprising. Because older adults with diabetes require more regular medical care than the general US adult population, we would expect a lower proportion of them to avoid health care. Similar to other researchers, we also found that factors such as cost (2,3), patient–provider communication (2,3,5), and education (2) were significantly associated with avoiding health care among Medicare beneficiaries with type 2 diabetes. However, contrary to previous findings, we found an association between race/ethnicity and avoidance of health care (2,5), and we did not find an association between income level (2) or sex (5) and health care avoidance. These findings, in part, are likely due to differences in study populations and settings. The differences, however, are worth noting and warrant further investigation.

Our study showed that Medicare beneficiaries with type 2 diabetes with lower educational attainment (<high school diploma and a high school diploma only vs >high school diploma) were more likely to avoid health care. Decision makers can use this information to identify Medicare beneficiaries with type 2 diabetes who are at risk of avoidance behavior. For example, tailoring materials (eg, educational resources) to account for potential low literacy may be important in ameliorating inequities among those without education beyond high school. This finding is important because lower health literacy and low levels of education have also been associated with poorer overall health (16). By extension, diabetes self-management education and support (DSMES) programs (10,17,18) can support Medicare beneficiaries with type 2 diabetes at risk of avoidance behavior, by tailoring materials to improve participants’ diabetes literacy and self-efficacy. For example, previous research evaluating the community-based peer-led DSMES found that participants with at-goal hemoglobin A_1c_ values significantly improved communication with physicians (18), a key factor that affects a person’s health care–seeking behaviors (2).

We also found a significant association between race/ethnicity of Medicare beneficiaries with type 2 diabetes and choices to avoid health care. This information can be used to tailor culturally sensitive interventions that can reduce the disparity in older adults of racial/ethnic minority populations who already experience racial/ethnic disparities in diabetes-associated care (19). Culturally and linguistically appropriate interventions that encourage engagement of racial/ethnic minority groups in diabetes-related care and management, which could reduce these disparities (20), especially among people choosing to avoid health care, are needed.

We found a significant association between choosing to avoid health care among Medicare beneficiaries with type 2 diabetes and the presence of health problems that should have been discussed with health care providers but were not. Medicare beneficiaries with type 2 diabetes who feel their concerns are not being addressed may be less inclined to participate in follow-up health care visits, particularly when they have time and cost concerns. Therefore, adapting interventions to patient–provider communication is important. Because Medicare beneficiaries with type 2 diabetes may expect health care providers to manage multiple health problems in 1 time-limited medical appointment, establishment of shared patient–provider expectations for a visit may bridge this gap. Providers need to express to their patients that they might not able to provide all necessary medical advices at a single appointment. Discussing the reasons for having multiple visits can be important for patients to have appropriate expectations. Previous research demonstrated that consistent high-quality patient–provider relationships are pivotal in optimizing health outcomes for people with chronic conditions such as diabetes (21,22).

Our findings indicated that Medicare beneficiaries with type 2 diabetes identified cost as a driving factor in decisions to avoid health care. This finding is consistent with other reports highlighting cost as an important consideration in health care decision making, even among insured people who have access to care. Wharam and colleagues reported delays in care seeking for macrovascular complications among employer-insured beneficiaries with a history of diabetes after a transition from low- to high-deductible health plans (23). Transitioning to a fixed retirement income with high out-of-pocket costs affects the medical care use of many Medicare beneficiaries (24). For example, the average annual out-of-pocket costs among Medicare beneficiaries with diabetes ($2,528 in 2020 US$) were approximately 30% higher than costs for beneficiaries without diabetes (25). Previous research reported that having to pay out-of-pocket expenses is a disincentive to using diabetes-associated preventive care (26). Therefore, policy discussions should involve the topic of cost sharing for Medicare beneficiaries, especially those with type 2 diabetes.

We did not find significant differences in avoiding health care by sex. However, the results of a recent survey from the Cleveland Clinic (27), focusing only on men, indicated that 65% of men may “wait as long as possible to see their doctor” when they have injuries or symptoms of a health condition. Although restricted to men, that survey provided additional insights into why men avoid physician visits, indicating that approximately 41% were told as children that “men don’t complain about health” (27). In addition, the same survey found that, among men who were not already having annual checkups, 61% would be more likely to participate in an annual check-up if it were “more convenient” (27). Future studies should focus on differences by sex.

Although our study did not find significant differences in avoiding health care by sex, the research at the Cleveland Clinic does provide insight into ways to help encourage people to have annual checkups. This finding is important in diabetes care because decisions to avoid health care may preclude early detection of diabetes, particularly among people with prediabetes. Additionally, avoiding health care increases the risk of undetected, preventable disease-related complications for people with established diabetes. Therefore, research that investigates sex differences in avoiding health care among Medicare beneficiaries with type 2 diabetes would be valuable.

Previous studies, with different populations and settings, found that health anxiety, which is “characterized by persistent preoccupation of having or acquiring a serious illness, misattribution of bodily symptoms and urge to seek medical advice in the absence of physical pathology” (28), was positively associated with increased health care use and greater medical expenditures (29,30). Interestingly, in our study, Medicare beneficiaries with type 2 diabetes who reported worrying about health more than others their age were more likely to avoid going to the doctor than people who did not report such worry. The reason for this health care decision-making process is unclear and warrants further investigation.

This study has several limitations. First, generalizability is limited because we focused only Medicare beneficiaries with type 2 diabetes and surveys were restricted to English or Spanish, potentially excluding those who do not speak these languages. Second, the cross-sectional analysis prevented us from drawing conclusions about cause and effect. Third, we used a dichotomous measure from a single survey item on our outcome of interest instead of using an open-ended question. Such a qualitative research approach, instead of our quantitative approach, could provide a more nuanced understanding of the complex reasons for avoiding health care. Fourth, our study was subject to recall bias (ie, relying on beneficiaries’ recollection of events). Fifth, information on annual household income was restricted to a single dichotomous variable of less than $25,000 and $25,000 or more.

Our findings suggest that further investigation is needed into the causes and economic implications of avoiding health care among Medicare beneficiaries with chronic conditions such as diabetes. Studies that can build on the results of our study are needed to develop screening tools for use by diabetes care providers to identify people at risk of avoiding health care. Additionally, there is a need to examine provider practices that support therapeutic patient–provider communication and effective relationship building. Finally, systems-level changes that limit factors associated with avoiding health care should be explored and implemented.

## Appendix Survey Questions That Measured Outcome Variable and Key Factors of Interest, Medicare Current Beneficiary Survey Public Use File 2016

**Table:**

Variable	Questions and Response Levels
Decisions to avoid or not seek health care	Please tell me whether each of the following statements is true or false. [You/sample person (SP)] will do just about anything to avoid going to the doctor. Response: true, false, refused, don’t know, inapplicable/missing.
Diabetes	Has a doctor ever told [you/(SP)] that (you/he/she) had any type of diabetes, including: sugar diabetes, high blood sugar, (borderline diabetes, prediabetes, or pregnancy-related diabetes/borderline diabetes, or prediabetes)? Response: yes, no, refused, don’t know, inapplicable/missing.
	Please tell me which type of diabetes the doctor said that [you have/(SP)] has Response: Type 1, Type 2, borderline, other, refused, inapplicable/missing.
Delayed seeking care because of costs	Since (LAST HF [Health Status and Functioning Questionnaire] MONTH YEAR), [have you/has (SP)] delayed seeking medical care because (you were/he was/she was) worried about the cost? Response: yes, no, refused, don’t know, inapplicable.
Dissatisfaction with ease of getting to providers	[Please tell me how satisfied you have been with . . .] the ease and convenience of getting to a doctor from where [you/(SP)] [live/lives]. Response: very satisﬁed, satisﬁed, dissatisﬁed, very dissatisﬁed, refused, don’t know, inapplicable/missing, no experience.
Dissatisfaction with information given by providers	[Please tell me how satisfied you have been with . . .] the information given to [you/you or (SP)] about what was wrong with [you/(SP)]. Response: very satisﬁed, satisﬁed, dissatisﬁed, very dissatisﬁed, refused, don’t know, inapplicable/missing, no experience.
Health problems that should have been discussed with providers were not	[You/(SP)] often [have/has] health problems that should be discussed but are not. Response: strongly agree, agree, neither agree nor disagree, disagree, strongly disagree, refused, don’t know, inapplicable.
Worried about health more than others your/their age	Please tell me whether each of the following statements is true or false. [You/(SP)] (worry/worries) about (your/his/her) health more than other people (your/his/her) age. [Is this statement true or false?] Response: true, false, refused, don’t know, inapplicable/missing.
